# Obesity and survival in operable breast cancer patients treated with adjuvant anthracyclines and taxanes according to pathological subtypes: a pooled analysis

**DOI:** 10.1186/bcr3572

**Published:** 2013-11-06

**Authors:** Bella Pajares, Marina Pollán, Miguel Martín, John R Mackey, Ana Lluch, Joaquín Gavila, Charles Vogel, Manuel Ruiz-Borrego, Lourdes Calvo, Tadeusz Pienkowski, Álvaro Rodríguez-Lescure, Miguel Angel Seguí, Olivier Tredan, Antonio Antón, Manuel Ramos, María del Carmen Cámara, César Rodríguez-Martín, Eva Carrasco, Emilio Alba

**Affiliations:** 1 Medical Oncology Department, Hospital Clínico Universitario Virgen de la Victoria, Málaga, Spain; 2 Epidemiology Nacional Center, Instituto de Salud Carlos III, Madrid, Spain; 3 Medical Oncology Department, Instituto de Investigación Sanitaria Gregorio Marañón, Universidad Complutense, Madrid, Spain; 4 Medical Oncology Department, Medical Cross Cancer Institute, Edmonton, AB, Canada; 5 Hematology-Oncology Department, Hospital Clínico Universitario de Valencia- INCLIVA Health Research Institute, University of Valencia, Valencia, Spain; 6 Medical Oncology Department, Instituto Valenciano de Oncología, Valencia, Spain; 7 Medical Oncology Department, Sylvester Comprehensive Cancer at Deerfield; Miller School of Medicine; Univ. of Miami- Deerfield Bch, Miami, FL, USA; 8 Medical Oncology Department, Hospital Virgen del Rocío, Sevilla, Spain; 9 Medical Oncology Department, Complejo Hospitalario Universitario de A Coruña, A Coruña, Spain; 10 Medical Oncology Department, European Health Center, Otwock, Poland; 11 Medical Oncology Department, Hospital General Universitario de Elche, Alicante, Spain; 12 Medical Oncology Department, Corporación Sanitaria Parc Taulí, Sabadell, Spain; 13 Medical Oncology Department, Centre Léon Bérard, Lyon, France; 14 Medical Oncology Department, Hospital Miguel Servet, Zaragoza, Spain; 15 Medical Oncology Department, Centro Oncológico de Galicia, A Coruña, Spain; 16 Spanish Breast Cancer Research Group, GEICAM, Madrid, Spain

## Abstract

**Introduction:**

Obesity is an unfavorable prognostic factor in breast cancer (BC) patients regardless of menopausal status and treatment received. However, the association between obesity and survival outcome by pathological subtype requires further clarification.

**Methods:**

We performed a retrospective analysis including 5,683 operable BC patients enrolled in four randomized clinical trials (GEICAM/9906, GEICAM/9805, GEICAM/2003–02, and BCIRG 001) evaluating anthracyclines and taxanes as adjuvant treatments. Our primary aim was to assess the prognostic effect of body mass index (BMI) on disease recurrence, breast cancer mortality (BCM), and overall mortality (OM). A secondary aim was to detect differences of such prognostic effects by subtype.

**Results:**

Multivariate survival analyses adjusting for age, tumor size, nodal status, menopausal status, surgery type, histological grade, hormone receptor status, human epidermal growth factor receptor 2 (HER2) status, chemotherapy regimen, and under-treatment showed that obese patients (BMI 30.0 to 34.9) had similar prognoses to that of patients with a BMI < 25 (reference group) in terms of recurrence (Hazard Ratio [HR] = 1.08, 95% Confidence Interval [CI] = 0.90 to 1.30), BCM (HR = 1.02, 0.81 to 1.29), and OM (HR = 0.97, 0.78 to 1.19). Patients with severe obesity (BMI ≥ 35) had a significantly increased risk of recurrence (HR = 1.26, 1.00 to 1.59, *P* = 0.048), BCM (HR = 1.32, 1.00 to 1.74, *P* = 0.050), and OM (HR = 1.35, 1.06 to 1.71, *P* = 0.016) compared to our reference group. The prognostic effect of severe obesity did not vary by subtype.

**Conclusions:**

Severely obese patients treated with anthracyclines and taxanes present a worse prognosis regarding recurrence, BCM, and OM than patients with BMI < 25. The magnitude of the harmful effect of BMI on survival-related outcomes was similar across subtypes.

## Introduction

Breast cancer (BC) is the most frequent invasive neoplasm in women. By 2015 the annual global incidence of BC is estimated at 1.6 million women [1]. The incidence in Europe alone will be over 450,000 new cases, resulting in 138,000 deaths [1]. Among others, obesity is a risk factor for several types of cancer, including BC [2], and has recently gained special relevance due in part to the dramatic increase in the prevalence of obesity observed worldwide. In fact, if current trends continue, over 50% of the world’s population will be obese by the year 2030 [3].

Recent cohort studies show that BC patients who are obese at diagnosis exhibit worse outcomes than their normal-weight counterparts in terms of probability of recurrence, contralateral BC, second primary malignancies, and overall mortality (OM) [4,5]. Whereas obese women may be at risk for being diagnosed at a more advanced stage of disease than other women, multivariate analyses confirm obesity as an independent prognostic factor for risk of recurrence and survival [5]. Additionally, recently published meta-analyses confirm a negative effect of obesity on BC prognosis [5-8]. These data, however, are mostly based on cohort studies with several limitations, such as sample heterogeneity regarding tumor stage, treatment received, follow-up period, and the use of self-reported height and weight data to calculate body mass index (BMI).

The association between obesity and prognosis is also explored in several clinical trials restricted to patients with early-stage BC. These studies were in patients receiving different treatments (for example, hormone therapy, chemotherapy protocols with/without anthracyclines) and reported conflicting results [9-15]. Although most of these studies showed an increased risk of death related to obesity, in the modern era of adjuvant anthracyclines and taxanes the association between BMI and disease recurrence and/or breast cancer mortality (BCM) requires further exploration together with an examination of the magnitude of such associations by pathological subtype, that is estrogen receptor (ER)/progesterone receptor (PR)-positive/human epidermal growth factor-2 (HER2)-negative, HER2-positive, triple-negative). The pooling of data from all patients participating in four high-quality randomized clinical trials with similar treatment and follow-up protocols offers a unique opportunity to assess the influence of BMI on clinical outcomes while minimizing unexplained variability commonly found in observational studies. Thus, the aim of this study was to assess the prognostic effect of BMI on recurrence, breast cancer mortality (BCM), and overall mortality (OM) among BC patients treated with adjuvant anthracyclines and taxanes, enrolled in four phase-III Spanish Breast Cancer Research Group (GEICAM) and Breast Cancer International Research Group (BCIRG) clinical trials (GEICAM/9906, GEICAM/9805, GEICAM/2003–02, and BCIRG 001) [16-20]. We also evaluated the prognostic role of obesity according to the different pathological BC subtypes as defined by immunohistochemistry.

## Methods

### Study design and participants

The cohort is comprised of 98% Caucasian women (reflecting the breast cancer patient population in Spain, as most patients included in this pooled analyses were from this country) from four randomized trials that evaluated systemic adjuvant chemotherapy (CT) based on anthracyclines and taxanes for lymph node-negative and lymph node-positive operable BC. These phase III trials accrued patients between 1996 and 2008 and evaluated the following CT protocols: 1) six cycles of docetaxel, doxorubicin, and cyclophosphamide (TAC) or fluorouracil, doxorubicin, and cyclophosphamide (FAC) for high-risk node-negative patients (GEICAM/9805) [17]; 2) six cycles of fluorouracil, epirubicin, and cyclophosphamide (FEC) or four cycles of FEC followed by weekly paclitaxel for node-positive patients (GEICAM/9906) [16]; 3) six cycles of TAC or FAC for node-positive patients (BCIRG 001) [18,19]; and 4) six cycles of FAC or four cycles of FAC followed by weekly paclitaxel for node-negative patients (GEICAM/2003-02) [20]. Chemotherapy was prescribed by actual body weight and was adjusted for obese patients in about 2% of patients (151 of 5,683 patients). After completing the CT treatment, tamoxifen, aromatase inhibitors, or the switching strategy (that is, tamoxifen followed by aromatase inhibitor) were administered for five years to patients with ER-positive and/or PR-positive tumors. Radiotherapy (RT) was mandatory after breast-conserving surgery and was administered after mastectomy, according to each institution’s guidelines. These trials were conducted before the approval of adjuvant trastuzumab therapy. Further details of the trial designs and findings have been reported elsewhere [16-20].

The Institutional Review Boards at the study sites approved these trials (see Additional file 1 for a list), and the participants provided written informed consent prior to randomization. For this analysis, we excluded patients who were randomized but not included in the corresponding trial (32 patients) and those who had incomplete height or weight data (9 women). The final cohort consisted of 5,683 patients.

### Procedure

The independent variable of interest, BMI, was calculated based on the height and weight of each participant at the start of the study. According to the World Health Organization definition, patients were classified into five BMI subgroups: underweight (<18.5 kg/m^2^), normal weight (18.5 to 24.9 kg/m^2^), overweight (25.0 to 29.9 kg/m^2^), obese (30.0 to 34.9 kg/m^2^) and severely obese (≥35.0 kg/m^2^) [21]. Due to the small number of underweight patients, the first two groups were combined and, thus, BMI <25 kg/m^2^ was used as the reference category. Other variables considered included: age, menopausal status, surgery type, histology type, histological grade, tumor size, nodal status, hormone receptor, and HER2 status. We included the category, HER2 unknown, due to the high number of participants with missing data in the GEICAM/9805 study. Regarding CT, the overall dose of each drug in the corresponding regimen was compared with the theoretical dose based on the patient’s estimated body weight using the real weight of the patient at enrollment [22]. As in other studies, we considered women who received a dose lower than 85% of the standard dose as undertreated [23]. Undertreatment was computed considering the initial dose (initial undertreatment) and the total amount of CT received (overall undertreatment). Toxicity was graded according to the National Cancer Institute Common Toxicity Criteria; we considered grade 3 and 4 adverse events to be clinically relevant [24].

We calculated the impact of obesity on different endpoints. Recurrence was defined as local, regional, or distant disease recurrence or the diagnosis of a second primary breast cancer, including contralateral breast tumors. *In situ* tumors were not considered as recurrence. BCM was defined as death due to BC, and overall mortality was defined as death from any cause. To guarantee the quality and homogeneity of the follow-up data all analyses were restricted to the first 10 years after recruitment.

### Statistical analysis

The Kaplan-Meier method was used to estimate survival, and the log-rank test was used to assess the possible differences between subgroups. Cox proportional hazards regression analysis was performed to assess the prognostic effect of BMI on recurrence, BCM, and OM [25]. Basic models were adjusted for the clinical trial from which data originated (study) - which also acted as a proxy for nodal involvement - and treatment regimen. Full models included additional adjustment for age, menopausal status, tumor size, histological grade, hormone receptor status, HER2 status, surgery type, and overall undertreatment (yes/no) as potential confounders. In addition, to explore the shape of the dose–response curve for BMI without assuming a linear relationship, natural splines were used in the full model, including four knots based on Harrell’s recommended percentiles, namely 5%, 35%, 65%, and 95% [26]. Finally, to test the consistency of the excess risk associated with higher BMI, subgroup analyses were conducted to estimate the effect of having BMI ≥35 compared to BMI <25 per category of the following variables: clinical trial, age, menopausal status, histological type, pathologic primary tumor size, nodal involvement, surgery type, hormone therapy (yes/no), undertreatment (yes/no), and pathological subtype. We report two-sided *P-*values, and *P* <0.05 was considered statistically significant. Statistical analyses were performed using STATA 12 (StataCorp LP, College Station, TX, USA).

## Results

The characteristics of the patients enrolled in each trial are provided as additional material (see Additional file 2). In total, 5,683 patients from four phase III trials with complete height and weight data were analyzed in the pooled data. The median follow-up time of patients who were alive at the time of this analysis was 93.4 months (range from 0.6 to 120). Table 1 describes sociodemographic and clinical characteristics of the sample and illustrates the relationship between patient characteristics and BMI category. For the sole purpose of simplifying the sample description here, we combined BMI into three categories. Using 30 kg/m^2^ as the cut off, 4,307 patients (75.8%) were classified as non-obese (BMI <30), 945 patients (16.6%) had BMI between 30 and 34.9 (obese) and 431 patients (7.8%) had BMI greater than or equal to 35 (severely obese). The median age was 48 years in the non-obese patients (range 42 to 56), 56 years among obese patients (range 49 to 62) and 55 years among the severely obese (range 49 to 62). Severely obese patients were more likely to be postmenopausal, to present lymph node positivity, and to be undertreated compared to non-obese patients. Additionally, severely obese patients were less likely to present with a tumor size <2 cm, undifferentiated tumors, or HER2-positive tumors. Even though the prevalence of undertreatment was low, as expected in clinical trials, there were significant differences in the doses (calculated as mg/m^2^) of CT between severely obese patients and non-obese patients. A higher proportion of severely obese patients received doses below 85% of the theoretical dose of CT compared with the non-obese patients (6.0% versus 2.4% in the first dose and 15.0% versus 7.1% considering the cumulative dose, *P* <0.001). Patients with severe obesity were as likely to present with severe adverse events (grades 3 to 4) as non-obese patients (42.0% versus 40.4%, *P* = 0.498).

**Table 1:** Distribution of variables by BMI category (non-obese, obese, and severely obese women)

	**BMI <30.0**	**BMI 30 to 34.9**	**BMI ≥ 35.0**	
	**Number (%)**	**Number (%)**	**Number (%)**	***P-*** **value**^**a**^
**Age at diagnosis, years**				<0.001
20 to 44	1,473 (34.2%)	138 (14.6%)	56 (13.0%)	
45 to 54	1,599 (37.1%)	284 (30.1%)	153 (35.5%)	
55 to 64	932 (21.6%)	364 (38.5%)	147 (34.1%)	
65 to 76	303 (7.0%)	159 (16.8%)	75 (17.4%)	
Median (percentiles 25 to 75)	48 (42 to 56)	56 (49 to 62)	55 (49 to 62)	
**Menopausal status**				<0.001
Postmenopausal	1,737 (40.3%)	631 (66.8%)	293 (68.0%)	
Premenopausal	2,570 (59.7%)	314 (33.2%)	138 (32.0%)	
**Histology**				0.730
Ductal	3,645 (84.6%)	788 (83.4%)	376 (87.2%)	
Lobulillar	385 (8.9%)	93 (9.8%)	32 (7.4%)	
Mixed	46 (1.1%)	12 (1.3%)	4 (0.9%)	
Others	230 (5.3%)	52 (5.5%)	19 (4.4%)	
Unknown	1 (0.0%)	0 (0.0%)	0 (0.0%)	
**Histological grade**				0.054
1	384 (8.9%)	81 (8.6%)	34 (7.9%)	
2	1,792 (41.6%)	416 (44.0%)	196 (45.5%)	
3	1,823 (42.3%)	381 (40.3%)	187 (43.4%)	
Unknown	308 (7.1%)	67 (7.1%)	14 (3.2%)	
**Pathologic primary tumor size**				<0.001
T1	2,192 (50.9%)	423 (44.8%)	179 (41.5%)	
T2	1,948 (45.2%)	485 (51.3%)	232 (53.8%)	
T3	167 (3.9%)	37 (3.9%)	20 (4.6%)	
**Nodes**				0.021
Negative	2,278 (52.9%)	487 (51.5%)	198 (45.9%)	
Positive	2,029 (47.1%)	458 (48.5%)	233 (54.1%)	
**Estrogen receptor**				0.647
Negative	1,398 (32.5%)	321 (34.0%)	137 (31.8%)	
Positive	2,899 (67.3%)	623 (65.9%)	291 (67.5%)	
Unknown	10 (0.2%)	1 (0.1%)	3 (0.7%)	
**Progesterone receptor**				0.199
Negative	1,654 (38.4%)	373 (39.5%)	149 (34.6%)	
Positive	2,576 (59.8%)	558 (59.0%)	276 (64.0%)	
Unknown	77 (1.8%)	14 (1.5%)	6 (1.4%)	
**Human epidermal growth factor-2 status**				0.030
Negative	3,036 (70.5%)	690 (73.0%)	329 (76.3%)	
Positive	656 (15.2%)	123 (13.0%)	51 (11.8%)	
Unknown	615 (14.3%)	132 (14.0%)	51 (11.8%)	
**Type of surgery**				0.071
Mastectomy	2,021 (46.9%)	405 (42.9%)	195 (45.2%)	
Conservative	2,286 (53.1%)	540 (57.1%)	236 (54.8%)	
**Hormonotherapy**				0.334
None	1,068 (24.8%)	256 (27.1%)	115 (26.7%)	
Yes	3,081 (71.5%)	664 (70.3%)	300 (69.6%)	
Unknown	158 (3.7%)	25 (2.6%)	16 (3.7%)	
**Adverse events (grades 3,4)**				0.498
No	2,565 (59.6%)	578 (61.2%)	250 (58.0%)	
Yes	1,742 (40.4%)	367 (38.8%)	181 (42.0%)	
**Initial under-treatment (in the first dose)**^**b**^				<0.001
No	4,205 (97.6%)	922 (97.6%)	405 (94.0%)	
Yes	102 (2.4%)	23 (2.4%)	26 (6.0%)	
Per type of treatment^c^				
Epirubicin <85%	5 (0.5%)	5 (2.3%)	4 (4.0%)	0.002
Cyclophosphamide <85%	12 (0.3%)	4 (0.4%)	8 (1.9%)	<0.001
Fluorouracil <85%	8 (0.2%)	4 (0.5%)	7 (2.2%)	<0.001
Doxorubicin <85%	4 (0.1%)	3 (0.4%)	7 (2.1%)	<0.001
Docetaxel <85%	24 (2.5%)	5 (2.4%)	6 (5.4%)	0.199
Paclitaxel <85%	62 (5.2%)	11 (4.2%)	7 (6.7%)	0.604
**Overall undertreatment**^**b**^				<0.001
No	3,903 (90.6%)	820 (86.8%)	360 (83.5%)	
Yes	404 (9.4%)	125 (13.2%)	71 (16.5%)	
Per type of treatment ^c^				
Epirubicin <85%	50 (5.5%)	15 (6.8%)	13 (13.1%)	0.011
Cyclophosphamide <85%	213 (4.9%)	49 (5.2%)	34 (7.9%)	0.032
Fluorouracil <85%	132 (3.9%)	25 (3.4%)	24 (7.5%)	0.005
Doxorubicin <85%	160 (4.7%)	40 (5.5%)	26 (7.8%)	0.040
Docetaxel <85%	117 (15.8%)	39 (18.9%)	10 (9.9%)	0.068
Paclitaxel <85%	159 (13.3%)	59 (22.6%)	26 (25.0%)	<0.001

^a^*P-*values computed excluding women without information in the corresponding variable; ^b^defined as total dose received lower than 85% of the theoretical dose prescribed; ^c^percentages including only women receiving the corresponding type of treatment. BMI, body mass index.

**Figure 1:**
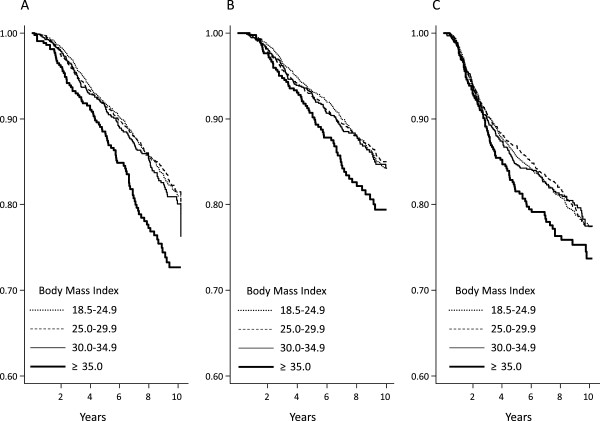
**Crude Kaplan-Meier curves for each survival outcome for each body mass index (BMI) category. (A)** Overall survival. The lower curve shows patients with BMI ≥35. Overall log-rank *P* = 0.002; log-rank comparing the curve for BMI ≥35 with the rest was <0.001. **(B)** Breast cancer survival. The lower curve shows patients with BMI ≥35. Overall log-rank *P* = 0.052; log-rank comparing the curve for BMI ≥35 with the rest = 0.006. **(C)** Recurrence-free survival. The lower curve shows patients with BMI ≥35. Overall log-rank *P* = 0.226; log-rank comparing the curve for BMI ≥35 with the rest = 0.040.

To illustrate the relationship between BMI and survival, we calculated crude Kaplan-Meier curves for recurrence-free survival, breast cancer survival and overall survival for each BMI category (Figure 1). In the Cox regression basic model (Table 2), obese patients’ outcomes were not significantly different from those of the reference group (BMI18.5 to 24.9), in terms of recurrence (Hazard Ratio [HR] 0.98, 95% CI 0.82, 1.17, *P* = 0.831), BCM (HR 1.04, 95% CI 0.83, 1.29, *P* = 0.757), or OM (HR 1.07, 95% CI 0.87, 1.3, *P* = 0.526). However, patients with severe obesity presented significantly worse outcomes in terms of BCM (HR 1.32, 95% CI 1.01, 1.72, *P* = 0.043) and OM (HR 1.47, 95% CI 1.16, 1.85, *P* = 0.001). Differences in recurrence did not attain statistical significance (HR 1.14, 95% CI 0.91, 1.42, *P* = 0.249) (Table 2 and Figure 1). Initial undertreatment was not related to any of the endpoints of interest, and was not considered in subsequent analyses.

**Table 2:** Multivariate Cox proportional hazards regression analysis of effect of BMI and each study variable on overall mortality, breast cancer mortality, and recurrence in basic models (adjusted for study and treatment regimen only)

		**Overall mortality**				**Breast cancer mortality**				**Recurrence**			
**Variable**	**Number**	**Events**	**HR**^**a**^	**95% CI**	***P-*** **value**	**Events**	**HR**	**95% CI**	***P-*** **value**	**Events**	**HR**	**95% CI**	***P-*** **value**
**Body mass index, k/m**^**2**^													
<18.5	66	11	1.08	0.59, 1.98	0.796	9	1.06	0.54, 2.06	0.864	12	0.88	0.49, 1.56	0.650
18.5 to 24.9	2,297	312	1.00			256	1.00			419	1.00		
25.0 to 29.9	1,944	265	0.99	0.84, 1.16	0.880	220	1.00	0.83, 1.20	0.984	348	0.95	0.83, 1.10	0.522
30.0 to 34.9	945	139	1.07	0.87, 1.30	0.526	111	1.04	0.83, 1.29	0.757	173	0.98	0.82, 1.17	0.831
≥35.0	431	91	1.47	1.16, 1.85	0.001	68	1.32	1.01, 1.72	0.043	95	1.14	0.91, 1.42	0.249
				*P for trend*	0.002			*P for trend*	0.074			*P for trend*	0.287
**Age at diagnosis, years**													
23 to 44	1,667	276	1.00			245	1.00			416	1.00		
45 to 54	2,036	228	0.66	0.55, 0.78	<0.001	187	0.61	0.50, 0.73	<0.001	296	0.54	0.47. 0.63	<0.001
55 to 64	1,443	202	0.92	0.77, 1.11	0.380	156	0.81	0.66, 0.99	0.036	231	0.66	0.56, 0.78	<0.001
65 to 76	537	112	1.41	1.13, 1.76	0.002	76	1.08	0.83, 1.39	0.583	104	0.81	0.65, 1.00	0.049
				*P for trend*	0.030			*P for trend*	0.655			*P for trend*	<0.001
**Menopausal status**													
Postmenopausal	2,661	421	1.00			322	1.00			461	1.00		
Premenopausal	3022	397	0.75	0.66, 0.86	<0.001	342	0.85	0.73, 0.98	0.031	586	1.05	0.93, 1.19	0.415
**Histology**													
Ductal	4,809	700	1.00			571	1.00			892	1.00		
Lobular	510	66	0.79	0.62, 1.02	0.072	52	0.76	0.57, 1.01	0.062	96	0.92	0.75, 1.14	0.446
Mixed	364	52	0.92	0.69, 1.22	0.561	41	0.89	0.65, 1.22	0.456	59	0.82	0.63, 1.07	0.150
Others	389	66	2.32	1.54, 3.49	<0.001	55	2.92	1.79, 4.75	<0.001	87	2.01	1.44, 2.83	0.000
**Histological grade**													
1	499	35	1.00			23	1.00			55	1.00		
2	2,404	277	2.09	1.47, 2.97	<0.001	217	2.51	1.63, 3.87	<0.001	382	1.84	1.39, 2.45	<0.001
3	2,391	440	3.70	2.62, 5.23	<0.001	369	4.77	3.13, 7.28	<0.001	523	2.82	2.13, 3.72	<0.001
Unknown	389	66	2.32	1.54, 3.49	<0.001	55	2.92	1.79, 4.75	<0.001	87	2.01	1.44, 2.83	<0.001
Pathologic primary tumor size													
T1	2,794	249	1.00			194	1.00			349	1.00		
T2	2,665	510	1.96	1.68, 2.28	<0.001	417	2.03	1.71, 2.41	<0.001	628	1.78	1.57, 2.04	<0.001
T3	224	59	2.14	1.61, 2.84	<0.001	53	2.39	1.76, 3.25	<0.001	70	1.98	1.53, 2.57	<0.001
				*P for trend*	<0.001			*P for trend*	<0.001			*P for trend*	<0.001
**Estrogen receptor/progesterone receptor**													
Both negative	1,502	300	1.00			256	1.00			349	1.00		
Any of them positive	4,132	503	0.49	0.43, 0.57	<0.001	394	0.45	0.38, 0.53	<0.001	679	0.59	0.51, 0.67	<0.001
Unknown	49	15	0.77	0.46, 1.31	0.338	14	0.82	0.48, 1.41	0.471	19	1.00	0.63, 1.59	0.998
**Human epidermal growth factor-2 status**													
Negative	4,055	518	1.00			407	1.00			660	1.00		
Positive	830	182	1.34	1.13, 1.59	0.001	153	1.42	1.18, 1.71	<0.001	223	1.38	1.18, 1.61	<0.001
Unknown	798	118	1.04	0.85, 1.28	0.690	104	1.17	0.94, 1 · 45	0.172	164	1.14	0.95, 1.36	0.157
**Type of surgery**													
Mastectomy	2,621	512	1.00			420	1.00			633	1.00		
Conservative	3,062	306	0.71	0.61, 0.82	<0.001	244	0.71	0.60, 0.83	<0.001	414	0.73	0.65, 0.83	<0.001
**Hormonotherapy**													
No	1,439	314	1.00			263	1.00			354	1.00		
Yes	4,045	451	0.42	0.36, 0.48	<0.001	356	0.39	0.33, 0.46	<0.001	622	0.51	0.45, 0.58	<0.001
Unknown	199	53	0.79	0.58, 1.06	0.112	45	0.77	0.56, 1.07	0.120	71	1.05	0.81, 1.37	0.692
**Initial undertreatment (first dose <85%)**													
No	5,532	648	1.00										
Yes	151	16	1.01	0.78,1.31	0.932	16	1.06	0.64, 1.75	0.820	20	0.84	0.54, 1.31	0.447
**Overall undertreatment (dose <85%)**													
None	5,083	725	1.00			599	1.00			943	1.00		
Epirubicine <85%	78	24	1.55	1.02, 2.36	0.040	20	1.56	0.98, 2.47	0.059	25	1.29	0.86, 1.93	0.226
Cyclophosphamide <85%	296	56	1.28	0.98, 1.69	0.074	41	1.13	0.82, 1.56	0.446	63	1.14	0.88, 1.47	0.323
Fluorouracil <85%	181	33	1.20	0.84, 1.70	0.316	23	1.01	0.66, 1.53	0.977	34	0.98	0.69, 1.38	0.888
Doxorubicin <85%	226	38	1.30	0.93, 1.81	0.119	27	1.12	0.76, 1.65	0.576	42	1.13	0.82, 1.54	0.451
Docetaxel <85%	176	32	1.08	0.74, 1.57	0.696	24	0.96	0.62, 1.47	0.846	39	1.04	0.74, 1.46	0.814
Paclitaxel <85%	244	27	1.43	0.94, 2.18	0.092	16	1.05	0.62, 1.78	0.863	30	1.09	0.74, 1.61	0.662

^a^Hazard ratio, 95% CI and *P*-value adjusted for study and treatment regimen.

In fully-adjusted models (Table 3), compared to the reference group (BMI 18.5 to 24.9) obesity remained a non-significant prognostic factor, but severe obesity independently increased the risk of recurrence (HR 1.25, 95% CI 0.99, 1.57, *P* = 0.052), BCM (HR 1.32, 95% CI 1.00, 1.74, *P* = 0.053), and OM (HR 1.35, 95% CI 1.06, 1.72, *P* = 0.016).

**Table 3:** Summary of multivariate Cox proportional hazards regression analysis of effect of BMI and each study variable on overall mortality, breast cancer mortality, and recurrence in full models

	**Overall mortality**			**Breast cancer mortality**			**Recurrence**		
**Variable**	**HR**^**a**^	**95% CI**	***P*** ***-*** **value**	**HR**	**95% CI**	***P*** ***-*** **value**	**HR**	**95% CI**	***P*** ***-*** **value**
**Body mass index, k/m2**									
<18.5	0.98	0.53, 1.79	0.945	0.95	0.48, 1.85	0.871	0.77	0.43, 1.37	0.377
18.5 to 24.9	1.00			1.00			1.00		
25.0 to 29.9	0.95	0.80, 1.12	0.549	1.01	0.84, 1.22	0.905	1.03	0.89, 1.19	0.728
30.0 to 34.9	0.98	0.79, 1.20	0.814	1.02	0.81, 1.29	0.860	1.07	0.89, 1.29	0.460
≥35	1.35	1.06, 1.72	0.016	1.32	1.00, 1.74	0.053	1.25	0.99, 1.57	0.052
**Age**									
Per 5 years	0.92	0.87, 0.97	0.003	0.86	0.81, 0.92	<0.001	0.84	0. 80, 0.89	<0.001
Menopausal status									
Postmenopausal	1.00			1.00			1.00		
Premenopausal	0.60	0.48, 0.74	<0.001	0.56	0.44, 0.71	<0.001	0.64	0.53, 0.77	<0.001
**Pathologic primary tumor size**									
T1	1.00			1.00			1.00		
T2	1.82	1.56, 2.32	<0.001	1.87	1.57, 2.22	<0.001	1.67	1.46, 1.91	<0.001
T3	1.88	1.40, 2.53	<0.001	2.07	1.51, 2.84	<0.001	1.71	1.31, 2.23	<0.001
**Histological grade**									
1	1.00			1.00			1.00		
2, 3, or unknown	1.26	1.07, 1.48	0.006	1.32	1.10, 1.58	0.003	1.24	1.07, 1.43	0.004
**Type of surgery**									
Mastectomy	1.00			1.00			1.00		
Conservative	0.82	0.70, 0.95	0.008	0.83	0.70, 0.98	0.028	0.84	0.74, 0.96	0.011
Estrogen receptor/progesterone receptor									
Both negative	1.00			1.00			1.00		
Any positive	0.56	0.48, 0.64	<0.001	0.51	0.44, 0.60	<0.001	0.66	0.57, 0.75	<0.001
Unknown	0.85	0.51, 1.44	0.558	0.89	0.52, 1.54	0.689	1.10	0.69, 1.75	0.699
**Human epidermal growth factor-2 status**									
Negative	1.00			1.00			1.00		
Positive	1.19	1.00, 1.42	0.048	1.23	1.01, 1.49	0.037	1.23	1.05, 1.44	0.009
Unknown	1.00	0.82, 1.23	0.965	1.11	0.89, 1.38	0.361	1.09	0.91, 1.30	0.335
**Undertreatment**									
None	1.00			1.00			1.00		
Epirubicine <85%	2.36	1.18, 4.72	0.015	3.06	1.47, 6.36	0.003	1.84	0.93, 3.63	0.078
Cyclophosph <85%	0.86	0.35, 2.13	0.74	0.93	0.34, 2.53	0.880	1.31	0.58, 2.95	0.519
Fluorouracil <85%	0.64	0.30, 1.34	0.235	0.45	0.19, 1.07	0.069	0.50	0.26, 0.99	0.046
Doxorubicin <85%	1.86	0.89, 3.88	0.097	1.86	0.82, 4.22	0.134	1.29	0.63, 2.64	0.478
Docetaxel <85%	0.84	0.46, 1.53	0.559	0.70	0.35, 1.40	0.315	0.75	0.44, 1.28	0.294
Paclitaxel <85%	1.37	0.90, 2.09	0.147	1.06	0.62, 1.81	0.824	1.15	0.78, 1.70	0.491

Models were adjusted for all the other variables in the table. ^a^Hazard ratio, 95% CI and *P-*value adjusted for study, treatment regimen and the rest of variables in the table.

**Figure 2:**
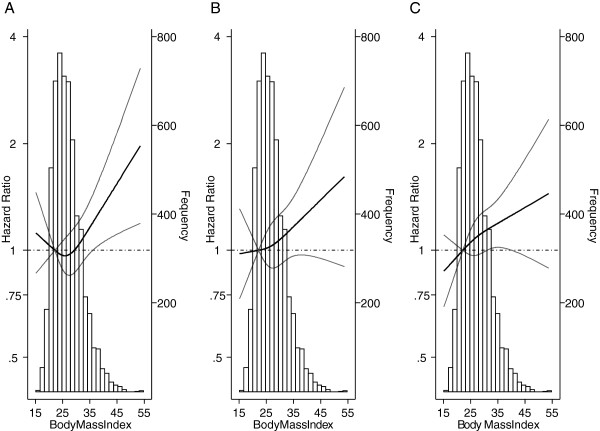
**Hazard ratio dose–response curve (dark line) and upper and lower limits of the confidence interval (lighter lines) for body mass index for each survival outcome. (A)** Overall Mortality. **(B)** Breast cancer mortality. **(C)** Recurrence. Estimates were adjusted for all the variables in the full model.

Figure 2 depicts the fully-adjusted HR dose–response curve (dark line) and upper and lower limits of the CI (lighter lines) for BMI for each of the three endpoints of interest. For OM, the dose–response curve showed higher HRs at both BMI extremes, but the 95% CIs did not include the one only at the right end of the curve, that is, for BMI ≥35. BCM and recurrence results also indicated a positive dose–response relationship, but the trend was not as pronounced and failed to reach statistical significance.

**Figure 3:**
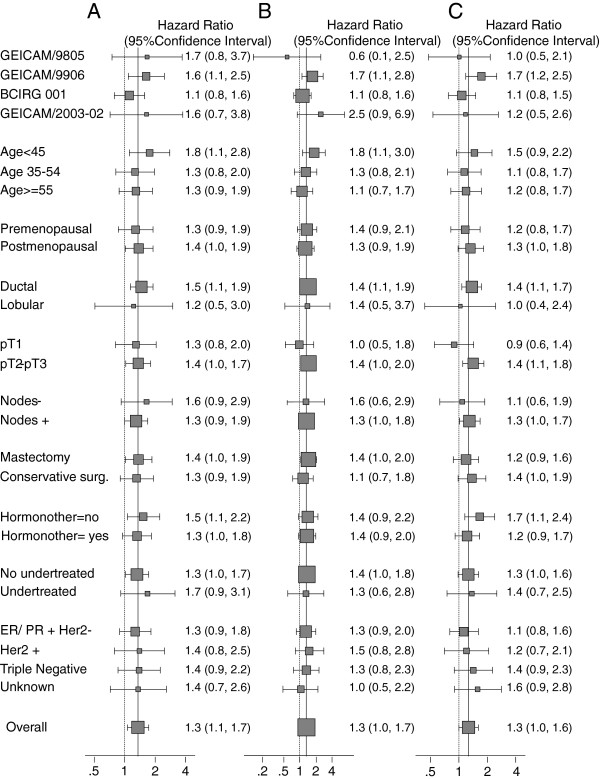
**Hazard ratios and 95% CI associated with severe obesity (body mass index ≥35) compared to the reference group (body mass index <25) for each survival outcome by the categories of each covariate. (A)** Overall mortality. **(B)** Breast cancer mortality. **(C)** Recurrence estimates were adjusted for all the variables in the full model. The size of the interval and the box are proportional to the amount of information available per stratum. GEICAM, Spanish Breast Cancer Research Group; BCIRG, Breast Cancer International Research Group; pT, pathologic primary tumor size; surg, surgery; HER2: human epidermal growth factor-2; ER, estrogen receptor; PR, progesterone receptor.

Figure 3 presents the HR and 95% CIs associated with severe obesity (BMI ≥35) for each survival outcome by the categories of each covariate, considering the different pathological BC subtypes. For these analyses, in order to increase our statistical power, BMI <25 was considered the reference category. These HR values were adjusted for the rest of the variables included in the full model. No statistically significant differences in the effect of severe obesity were observed per category of the other explanatory variables, but the effect seemed to be more pronounced in younger women (age <45 years). Furthermore, the magnitude of the negative effect of severe obesity on survival outcomes was similar across the three BC subtypes (ER/PR-positive/HER2-negative, HER2-positive, triple-negative).

## Discussion

In this retrospective survival analysis of pooled data from four large, well-conducted, randomized phase III trials of adjuvant CT, severe obesity (BMI ≥35) was significantly associated with worse survival outcomes (OM and BCM) and a higher risk of disease recurrence. Additionally, the magnitude of the negative effect of BMI on survival was similar across pathological subtypes (ER/PR-positive/HER2-negative, HER2-positive, triple-negative). However, obese patients (BMI 30.0 to 34.9) displayed similar prognosis to patients with BMI below 25.0, not unexpected given that most studies of women participating in clinical trials show a relatively modest effect of obesity on prognosis compared with population-based studies. A recent multi-analysis [8] concluded that there was a (non-statistically significant) tendency to report higher prognostic effects of obesity in BC patients in observational studies than in randomized controlled trials (RCTs). In this regard, our study supports the harmful prognostic effect of severe obesity among operable BC patients treated with the most up-to-date CT schedules based on anthracyclines and taxanes.

To the best of our knowledge, this is the largest analysis of RCTs examining obesity as a prognostic factor in operable BC patients treated with anthracyclines and taxanes, and adjusting for hormone receptor and HER2 status. It is also the second study to confine significant prognostic effects to severely obese patients, supporting previous work by Dignam and colleagues [27]. In a study conducted by Berclaz *et al*., data from 6,792 BC patients randomized in seven trials of the International Breast Cancer Study Group from 1978 to 1993 were analyzed [9]. These trials included observation (no adjuvant therapy), endocrine therapy (tamoxifen), or cyclophosphamide, methotrexate and fluorouracil (CMF)-based CT. Their results showed that BMI independently predicted OM (*P* = 0.03) but not risk of recurrence (*P* = 0.12). Azambuja *et al*. analyzed 2,887 node-positive BC patients included in the BIG 02–98 trial who were treated with a CT regimen based on anthracyclines. In this study, obesity emerged as an independent unfavorable prognostic factor for OM (*P* = 0.008) and recurrence (*P* = 0.04) [11]. A retrospective analysis of 4,077 women from the National Surgical Adjuvant Breast and Bowel Project clinical trials with ER-negative, node-negative BC, treated with several CT regimens with or without anthracyclines, showed higher mortality only among severely obese patients but not among obese patients [27]. Also our results are consistent with those obtained by Sparano *et al*. in the ECOG 1199 study [12] in which there was also a correlation between increased BMI and poorer disease-free survival and overall survival. However, in our study the correlation between BMI >35 and worse prognosis was observed in all pathological subtypes, whereas in the ECOG 1199 this effect seemed to be limited to ER-positive patients. These differences may be due to a different definition of ER-positive status (>10% in ECOG 1199 versus >1% in our studies). Furthermore, the percentage of obese patients was lower in our study (16.6 versus 36.6% respectively) and we did not include black patients. This is relevant because the ECOG 1199 has shown a significant association between black race, increased frequency of obesity and a worse prognosis in ER-positive patients [28].

Certain characteristics of our study may explain the differences between our results and previous work. First, the homogeneity of our sample, which included only patients with low comorbidity, could partially explain the stronger prognostic role of obesity observed in population-based studies than in our study. Such differences in effect may be attributed to other conditions such as diabetes, metabolic syndrome, and other chronic diseases that are associated both with obesity and with poorer prognoses. Because of the criteria used in clinical trials, obese patients with impaired health due to these conditions have a lower probability of being included in a clinical trial. Second, the assessment necessary to establish patient eligibility for a clinical trial may result in more accurate staging and consequently less confounding between obesity and delayed diagnosis often observed in obese women. Third, our results are based on a large sample size of more than 5,600 patients. Pooled analyses have shown that risk estimates based on retrospective analysis of clinical trials often decline as the sample size increases [9-11]. Finally, all our patients were treated homogeneously with anthracycline-based CT, such an effective treatment that it may have partly counteracted the adverse prognostic effect of obesity when compared to the obesity effect found in population and cohort studies, or even in clinical trials with suboptimal CT schedules like CMF. It should also be anticipated that undertreatment is less frequent when patients are treated within the context of a clinical trial.

In fact, undertreatment may also contribute to poorer survival rates in obese BC patients [22,29]. Several studies have shown that obese BC patients are more likely to receive reduced doses of CT compared to normal-weight women [22,29,30]. Obese patients treated with CMF-based CT were significantly more likely to receive a lower CT dose for the first course (<85% of expected dose) than those with normal or intermediate BMI values (39% versus 16%, *P* <0.0001) and this undertreatment was associated with a significantly worse outcome for ER-negative patients [27]. However, several studies found that obese women receiving full doses of CT did not experience more toxicity than normal-weight patients [22,30,31]. In our study, although undertreatment was generally low, severely obese patients were also more likely to be undertreated, despite the prevalence of serious adverse events being similar to that observed in non-obese patients. Even in the context of a clinical trial, severely obese patients were almost three times more likely to receive suboptimal doses from the start, and this insufficient dose was maintained in subsequent cycles in more than 90% of cases. Oncologists should be aware of the importance of prescribing full weight-based doses in agreement with the recommendations of the American Society of Clinical Oncology [22].

The pathways involved in the relationship between obesity and BC outcomes remain unclear, but obesity is known to affect several hormones and growth factors that are potentially associated with BC [32]. Consequently, obese patients may have elevated tumor cell proliferation and metastasis due to undefined adipokine effects on tumor cells [33]. For instance, hyperinsulinism has been correlated with BMI, recurrence, and BCM, regardless of hormone receptor status [34]. The pro-angiogenic and pro-inflammatory adipokines such as leptin, IL6, TNF-α, and vascular endothelial growth factor (VEGF), secreted by fat tissue, are commonly elevated in obese patients [35,36]. As BMI is directly related to circulating estrogen levels, the production of estrogens by adipose tissue has also been postulated as a factor in the more biologically aggressive ER-positive tumors in postmenopausal women [37]. Also higher rates of angiolymphatic invasion among obese women with breast cancer may contribute to their poorer outcomes, as described by Gillespie *et al*. in their retrospective series [38].

Several studies have evaluated the role of obesity as a risk factor for BC development according to different BC subtypes [39-42]. However, data on the role of obesity on patient prognosis according to these subtypes are scarce. A recent meta-analysis found no evidence of the prognostic role of obesity varying by hormone receptor status [7], although HER2 receptor data were not available until recently. In fact, a study on 4,770 operable BC patients treated with a CT regimen based on anthracyclines and taxanes reported a worse outcome specifically in hormone receptor-positive/HER2-negative disease patients, but not among patients with HER2-positive or triple-negative disease [12]. In contrast, our analyses failed to detect a difference in the observed prognostic role of severe obesity by subtype.

Although the shortcomings of our study are not uncommon in retrospective analyses of clinical trials, our results should be interpreted in the context of the study limitations. BMI was measured only at the beginning of follow up and further changes were not considered, in part due to the difficulty in assessing changes influenced by CT/hormone treatment side effects. For the analysis by tumor subtype, hormone receptor positivity was assessed in each trial under the available criteria at that moment, and the lack of information on HER2 in the GEICAM/9805 trial substantially decreases the statistical power of the analyses. Major strengths of this study include a large sample size (n >5,600), precise BMI information at baseline, and a standardized CT treatment based on the most active drugs in the adjuvant setting available at that time (anthracyclines and taxanes).

In summary, this study shows that obesity (BMI 30.0 to 34.9) is not associated with worse survival outcomes in operable BC patients treated with anthracycline- and taxane-based CT. Severely obese patients (BMI ≥35.0), however, present an increased risk of recurrence, BCM, and OM compared to patients with BMI <25.0. Further, the magnitude of the negative effect of BMI on survival outcomes was similar across pathological BC subtypes.

## Conclusions

Based on a large retrospective analysis of four randomized clinical trials of operable BC patients treated with anthracyclines and taxanes, and adjusting for hormone receptor and HER2 status, we found that severe obesity, but not obesity, emerged as a statistically and clinically significant unfavorable prognostic factor regardless of BC subtype. These findings support further basic and clinical research on the mechanisms of the association between severe obesity and survival outcomes in BC patients in order to maximize treatment benefits among these patients.

## Abbreviations

BC: Breast cancer; BCIRG: Breast Cancer International Research Group; BCM: Breast cancer mortality; BMI: Body mass index; CMF: Cyclophosphamide, methotrexate, fluorouracil; CT: Chemotherapy; ER: Estrogen receptor; FAC: Fluorouracil, doxorubicin, cyclophosphamide; FEC: Fluorouracil, epirubicin, and cyclophosphamide; GEICAM: Spanish Breast Cancer Research Group; HER2: Human epidermal growth factor-2; HR: Hazard ratio; OM: Overall mortality; PR: Progesterone receptor; RCT: Randomized controlled trial; RT: Radiotherapy; TAC: Docetaxel, doxorubicin, cyclophosphamide; TNF: Tumor necrosis factor.

## Competing interests

All authors declare that they have no competing interests.

## Authors’ contributions

BP, MP, and EA conceived the study. MM, JRM, AL, JG, CV, MRB, LC, TP, ARL, MAS, OT, AA, MR, MCC, CRM, EC and EA contributed to data collection. MP analyzed the data. BP and MP interpreted the results and wrote the first draft. All authors contributed to the writing and editing of the manuscript and approved the final version. EA had full access to all the data in the study and had final responsibility for the decision to submit for publication.

## Supplementary Material

Additional file 1 List of Institutional Review Boards. Click here for file

Additional file 2 Characteristics of women enrolled in the four clinical trials. Click here for file
