# Supplementary material for: Obesity and survival in operable breast cancer patients treated with adjuvant anthracyclines and taxanes according to pathological subtypes: a pooled analysis
Source: Breast Cancer Res. 2013 Nov 6;15(6):R105. doi: 10.1186/bcr3572 (PMC3978725; doi:10.1186/bcr3572)
Supplement: Additional file 1 — List of Institutional Review Boards. [file bcr3572-S1.pdf]

Additional File: List of Institutional Review Boards

The studies were approved by the corresponding Institutional Review Boards of the following participants Centers:

GEICAM/9906

SPAIN: CENTRE HOSPITAL DE MANRESA, CENTRO ONCOLÓGICO DE GALICIA, CLÍNICA ONCLOLÓGICA CORACHÁN, COMPLEJO HOSPITAL CIUDAD REAL, COMPLEJO HOSPITAL ORENSE, COMPLEJO HOSPITAL UNIVERSITARIO A CORUÑA, COMPLEJO HOSPITALARIO DE JAÉN, CONSORCI HOSPITAL PARC TAULÍ, CONSORCI SANITARI DE TERRASA, FUNDACIÓN HOSPITAL ALCORCÓN, HOSPITAL ARNAU DE VILANOVA, HOSPITAL CLINIC I PROVINCIAL, HOSPITAL CLÍNICO UNIVERSITARIO DE VALENCIA, HOSPITAL CLÍNICO UNIVERSITARIO SAN CARLOS, HOSPITAL COMARCAL DE BARBASTRO, HOSPITAL DE CABUEÑES, HOSPITAL DE CRUCES, HOSPITAL DE ELDA, HOSPITAL DE LA RIBERA, HOSPITAL DE LEÓN, HOSPITAL DE NAVARRA, HOSPITAL DE TXAGORRITXU, HOSPITAL DEL ESPÍRITU SANTO, HOSPITAL DEL MAR, HOSPITAL DONOSTIA, HOSPITAL DR. NEGRÍN, HOSPITAL DR. PESET, HOSPITAL G. DE ALICANTE, HOSPITAL GENERAL DE ELCHE, HOSPITAL GENERAL DE GUADALAJARA, HOSPITAL GENERAL DE JEREZ, HOSPITAL GENERAL DE MÓSTOLES, HOSPITAL GENERALUNIVERSITARIO DE VALENCIA, HOSPITAL GERMANS TRÍAS I PUJOL, HOSPITAL INSULAR, HOSPITAL JUAN RAMÓN JIMÉNEZ, HOSPITAL LUCUS AUGUSTI, HOSPITAL MORALES MESSEGUER, HOSPITAL MUNICIPAL DE BADALONA, HOSPITAL MUTUA TERRASA, HOSPITAL PROVINCIAL DE CÓRDOBA, HOSPITAL PROVINCIAL DE LA MISERICORDIA, HOSPITAL PROVINCIAL DE ZAMORA, HOSPITAL PUERTA DE HIERRO, HOSPITAL PUERTA DEL MAR, HOSPITAL PUERTO DE SAGUNTO, HOSPITAL RAMÓN Y CAJAL, HOSPITAL RÍO CARRIÓN, HOSPITAL SANT JOAN DE REUS, HOSPITAL UNIVERSITARIO DE CANARIAS, HOSPITAL UNIVERSITARIO DE SALAMANCA, HOSPITAL UNIVERSITARIO DE VALLADOLID, HOSPITAL UNIVERSITARIO LA FE, HOSPITAL UNIVERSITARIO MARQUÉS DE VALDECILLA, HOSPITAL UNIVERSITARIO MIGUEL SERVET, HOSPITAL UNIVERSITARIO PUERTO REAL, HOSPITAL UNIVERSITARIO SAN CECLIO, HOSPITAL UNIVERSITARIO VIRGEN DE LA ARRIXACA, HOSPITAL UNIVERSITARIO VIRGEN DE LA VICTORIA, HOSPITAL UNIVERSITARIO VIRGEN DEL ROCÍO, HOSPITAL VIRGEN DE LA SALUD, HOSPITAL VIRGEN DE LOS LIRIOS, HOSPITAL COMPLEJO UNIVERSITARIO LOZANO BLESA, INSTITUTO VALENCIANO DE ONCOLOGÍA, ONKOLOGIKOA.

GEICAM/2003-02

SPAIN: CENTRO ONCOLOGICO DE GALICIA, CLÍNICA PUERTA DE HIERRO, COMPLEJO HOSPITALARIO JUAN CANALEJO, CONSORCI SANITARI DE TERRASA, CONSORCI SANITARI PARC TAULI, FUNDACIÓN HOSPITAL ALCORCÓN, HOSPITAL ALTHAIA MANRESA, HOSPITAL ARNAU DE VILANOVA VALENCIA, HOSPITAL CARLOS HAYA, HOSPITAL CIUDAD DE JAÉN, HOSPITAL CIUDAD REAL, HOSPITAL CLINIC I PROVINCIAL, HOSPITAL CLÍNICO UNIVERSITARIO LOZANO BLESA, HOSPITAL CLÍNICO UNIVERSITARIO SAN CARLOS, HOSPITAL CLÍNICO UNIVERSITARIO VALENCIA, HOSPITAL CLÍNICO UNIVERSITARIO VIRGEN DE LA VICTORIA, HOSPITAL DE BARBASTRO, HOSPITAL DE BASURTO, HOSPITAL DE ELDA, HOSPITAL DE LA PRINCESA, HOSPITAL DE LA RIBERA, HOSPITAL DE MADRID-MONTEPRÍNCIPE, HOSPITAL DE MÓSTOLES, HOSPITAL DE SALAMANCA, HOSPITAL DE VALME, HOSPITAL DE VIC, HOSPITAL DEL ESPIRITU SANTO, HOSPITAL DEL MAR, HOSPITAL DONOSTIA, HOSPITAL GENERAL DE ALBACETE, HOSPITAL GENERAL DE ELCHE, HOSPITAL GENERAL RIO CARRION, HOSPITAL GENERAL UNIVERSITERIO DE ALICANTE, HOSPITAL GENERAL UNIVERSITARIO DE GUADALAJARA, HOSPITAL GENERAL UNIVESITARIO J.M. MORALES MESEGUER, HOSPITAL GENERAL YAGÜE, HOSPITAL GERMANS TRIAS I PUJOL, HOSPITAL INSULAR DE LAS PALMAS, HOSPITAL JEREZ DE LA FRONTERA, HOSPITAL LA FE, HOSPITAL MADRID - SANCHINARRO (CIOCC), HOSPITAL MARQUÉS DE VALDECILLA, HOSPITAL MIGUEL SERVET, HOSPITAL MUTUA TERRASA, HOSPITAL

P. DE CASTELLÓN, HOSPITAL P. UNIVERSITARIO REINA SOFIA, HOSPITAL POLICLÍNICO VIGO S.A. POVISA, HOSPITAL PUERTA DEL MAR, HOSPITAL RAMÓN Y CAJAL, HOSPITAL RGUEZ.CHAMORRO ZAMORA, HOSPITAL RUBER INTERNACIONAL, HOSPITAL SANT JOAN DE REUS, HOSPITAL STA. CREU I SANT PAU, HOSPITAL STA. MARÍA NAI, HOSPITAL TXAGORRITXU, HOSPITAL UNIVERSITARIO DE CANARIAS, HOSPITAL UNIVERSITARIO DOCE DE OCTUBRE, HOSPITAL UNIVERSITARIO LA PAZ, HOSPITAL UNIVERSITARIO VIRGEN DE LA ARRIXACA, HOSPITAL VIRGEN DE LA LUZ, HOSPITAL VIRGEN DE LA SALUD, HOSPITAL VIRGEN DE LOS LIRIOS, HOSPITAL VIRGEN DEL ROCIO, HOSPITAL XERAL CALDE DE LUGO, INSTITUT CATALÁ D'ONCOLOGÍA "JOSEP TRUETA" GIRONA, INSTITUTO DE ONCOLOGÍA CORACHÁN, INSTITUTO ONCOLÓGICO DE GUIPUZCOA, INSTITUTO VALENCIANO DE ONCOLOGÍA.

GEICAM/9805

GERMANY: KLINIK UND POLIKLINIK GYNÄKOLOGIE MARTIN-LUTHER-UNIVERSITÄTSKLINIK, KLINIKUM MANNHEIM FRAUENKLINIK, ST. MARIEN-KRH FRAUENKLINIK, UNIVERSITÄTS FRAUENKLINIK GYNÄKOLOGISCHE ONKOLOGIE KLINIKUM, **POLAND:** WIELKOPOLSKIE CENTRUM ONKOLOGI, WOJEWODZKI SZPITAL SPECJALISTYCZNY, **SPAIN:** CENTRO ONCOLÓGICO DE GALICIA, CLINIC I PROVINCIAL, CLÍNICA CORACHÁN, COMPLEJO HOSPITALARIO ARQUITECTO MARCIDE, COMPLEJO HOSPITALARIO DE JAÉN, COMPLEJO HOSPITALARIO DE LA CORUÑA, COMPLEJO HOSPITALARIO DE PONTEVEDRA, COMPLEJO HOSPITALARIO SANTA MARÍA NAI, COMPLEJO HOSPITALARIO XERAL CALDE DE LUGO, COMPLEJO HOSPITALARIO XERAL CÍES DE VIGO, CONSORCI SANITARI DE TERRASA, CORPORACION SANITARIA PARC TAULÍ, FUNDACIÓN HOSPITAL ALCORCÓN, GERMANS TRIAS I PUJOL, HOPSITAL DE LA RIBERA, HOSPITAL PUERTA DEL MAR, HOSPITAL ARNAU DE VILANOVA LÉRIDA, HOSPITAL ARNAU DE VILANOVA VALENCIA, HOSPITAL CLÍNICO DE VALENCIA, HOSPITAL CLÍNICO SAN CECILIO, HOSPITAL COMARCAL DE BARBASTRO, HOSPITAL DE BASURTO, HOSPITAL GENERAL DE ALBACETE, HOSPITAL GENERAL DE ELCHE, HOSPITAL GENERAL DE ELDA, HOSPITAL GENERAL DE PALENCIA RÍO CARRIÓN, HOSPITAL GENERAL DE VIC, HOSPITAL GENERAL UNIVEERSITARIO DE ALICANTE, HOSPITAL LA FE DE VALENCIA, HOSPITAL NTRA. SRA. DE ARÁNZAZU, HOSPITAL PROVINCIAL RODRÍGUEZ CHAMORRO, HOSPITAL RUBER INTERNACIONAL, HOSPITAL SANT JOAN REUS, HOSPITAL UNIVERSITARIO CLÍNICO SAN CARLOS, HOSPITAL UNIVERSITARIO DE LA PRINCESA, HOSPITAL UNIVERSITARIO DE SALAMANCA, HOSPITAL UNIVERSITARIO INSULAR LAS PALMAS, HOSPITAL UNIVERSITARIO LOZANO BLESA, HOSPITAL UNIVERSITARIO MARQUÉS DE VALDECILLA, HOSPITAL UNIVERSITARIO MIGUEL SERVET, HOSPITAL UNIVERSITARIO PUERTO REAL, HOSPITAL UNIVERSITARIO REINA SOFÍA, HOSPITAL UNIVERSITARIO VIRGEN DEL ROCÍO, HOSPITAL UNIVERSTIARIO RAMÓN Y CAJAL, HOSPITAL VIRGEN BLANCA DE LEÓN, HOSPITAL VIRGEN DE LA LUZ, HOSPITAL VIRGEN DE LA VICTORIA, HOSPITAL VIRGEN DE LOS LIRIOS, INSTITUTO VALENCIANO DE ONCOLOGÍA.

BCIRG 001

ARGENTINA: HOSPITAL BRITANICO DE BUENOS AIRES; INSTITUTO DE ONCOLOGIA ANGEL H ROFFO, BUENOS AIRES; HOSPITAL ITALIANO BUENOS AIRES, **AUSTRIA:** HOSPITAL RUDOLFSTIFTUNG WIEN **BRAZIL** CAISM-CAMPINAS UNIVERSITY, **CANADA:** BRITISH COLUMBIA CANCER AGENCY, VANCOUVER ISLAND CANCER CENTRE; LAKERIDGE HEALTH OSHAWA; HÔTEL DIEU DE LEVIS; HOPITAL FLEURIMONT, SHERBROOKE; BRITISH COLUMBIA CANCER AGENCY, VANCOUVER; LETHBRIDGE CANCER CENTRE; HÔPITAL DU SACRÉ-COEUR MONTRÉAL; SOUTHLAKE REGIONAL HEALTH CENTRE; HÔTEL DIEU DE MONTREAL; MONCTON HOSPITAL; BRAMPTON CIVIC HOSPITAL; WOMEN'S COLLEGE HOSPITAL TORONTO; OTTAWA HOSPITAL REGIONAL CANCER CENTRE; SAULT AREA HOSPITALS; HOPITAL NOTRE-DAME MONTREAL, **CZECH REPUBLIC:** THOMAYER TEACHING HOSPITAL PRAGUE;

UNIVERSITY HOSPITAL PLZEN, **EGYPT:** CAIRO ONCOLOGY CENTRE; MISR INTERNATIONAL HOSPITAL CAIRO, **GERMANY:** UNIVERSITATKLINIKUM ESSEN, **GREECE:** UNIVERSITY HOSPITAL OF HERAKLION **HUNGARY** SZENT MARGIT HOSPITAL, BUDAPEST, **ISRAEL:** RABIN MEDICAL CENTRE PETACH-TIKVA; TEL-AVIV SOURASKY MEDICAL CENTRE; CARMEL MEDICAL HOSPITAL HAIFA, **POLAND:** CENTRUM MEDYCZEN SOPMED SOPOT, **PORTUGAL:** HOSPITAL CONDES CASTRO DE GUIMARAES CASCAIS; HOSPITAL SAO BERNARDO SETUBAL, **SLOVAKIA:** NATIONAL CANCER INSTITUTE BRATISLAVA, **SOUTH AFRICA:** JOHANNESBURG GENERAL HOSPITAL, **SPAIN:** HOSPITAL CLÍNICO VIRGEN DE LA VICTORIA, MALAGA; HOSPITAL DONOSTIA SAN SEBASTIAN; HOSPITAL MIGUEL SERVET ZARAGOZA; HOSPITAL UNIVERSITARIO REINA SOFIA, CORDOBA; HOSPITAL UNIVERSITARIO DE GUADALAJARA; HOSPITAL CLINICO SAN CECILIO GRANADA; FUNDACION JIMENEZ DIAZ MADRID; HOSPITAL MARQUES DE VALDECILLA SANTANDER; COMPLEXO HOSPITALARIO DE SANTIAGO DE COMPOSTELA; HOSPITAL UNIVERSITARIO LA FE VALANCIA; HOSPITAL INSULAR DE GRAN CANARIA LAS PALMAS; HOSPITAL SANT JOAN REUS, **SWEDEN:** RADIUMHEMMET, KAROLINSKA HOSPITAL STOCKHOLM, **UK:** IPSWICH HOSPITAL, **URUGUAY:** HOSPITAL CENTRAL DE LA FUERZAS ARMADAS, MONTEVIDEO; HOSPITAL DE CLINICAS MONTEVIDEO, **USA:** HEMATOLOGY–ONCOLOGY OF KNOXVILLE, TN; ST LUKE'S MOUNTAIN STATES TUMOR INSTITUTE, ID; THE CENTER FOR HEMATOLOGY-ONCOLOGY BOCA RATON, FL; BAY AREA ONCOLOGY, FL; UCLA, CA; JACKSON ONCOLOGY ASSOCIATES, MS; HEMATOLOGY–ONCOLOGY ASSOCIATES OF THE TREASURE COAST, FL; SARAH CANNON RESEARCH INSTITUTE, TN; CAROLINAS HEMATOLOGY–ONCOLOGY ASSOCIATES, NC; DUKE UNIVERSITY MEDICAL CENTER, NC; CANCER CENTERS CAROLINAS, SC; FAIRFAX–NORTHERN VIRGINIA HEMATOLOGY–ONCOLOGY, VA; CENTRAL GEORGIA CANCER CARE, GA; NEW ENGLAND MEDICAL CENTER, MA; PHOEBE CANCER CENTER, GA; THE CANCER CENTER, PROVIDENCE HOSPITAL, AL; MEDICAL ONCOLOGY & HEMATOLOGY CONSULTANTS, TN; ONCOLOGY AND HEMATOLOGY ASSOCIATES, FL; BAY AREA ONCOLOGY, FL; BAPTIST HOSPITAL EAST TENNESSEE, TN; ONCOLOGY CONSULTANTS, TX.
