# Supplementary material for: Obesity and survival in operable breast cancer patients treated with adjuvant anthracyclines and taxanes according to pathological subtypes: a pooled analysis
Source: Breast Cancer Res. 2013 Nov 6;15(6):R105. doi: 10.1186/bcr3572 (PMC3978725; doi:10.1186/bcr3572)
Supplement: Additional file 2 — Characteristics of women enrolled in the four clinical trials. [file bcr3572-S2.pdf]

Additional Table: Characteristics of women enrolled in the four clinical trials

Variables	Study GEICAM/9805 N=1060	Study GEICAM/9906 N=1246	Study BCIRG001 N=1491	Study GEICAM/2003-02 N=1925
Recruitment period	Jun 1999 to Mar 2003	Nov 1999 to Jun 2002	Apr 1997 to May 1999	Sep 2003 to May 2008
Age at Diagnosis				
23-44	312 (29.4%)	365 (29.3%)	482 (32.3%)	519 (27. 0%)
45-54	356 (33.6%)	422 (33.9%)	577 (38.7%)	694 (36.1%)
55-64	308 (29.1%)	279 (22.4%)	343 (23.0%)	523 (27.2%)
65-76	84 (7.9%)	180 (14.4%)	89 (6.0%)	189 (9.8%)
Mean (SD)	*50.3 (10.1)*	*51.1 (10.8)*	*49.1 (9.4)*	*50.9 (9.96)*
Menopausal Status				
Postmenopausal	487 (45.9%)	567 (45.5%)	655 (43.9%)	971 (50.4%)
Premenopausal	573 (54.1%)	679 (54.5%)	836 (56.1%)	954 (49.6%)
Histology				
Ductal	908 (85.7%)	1060 (85.1%)	1227 (82.3%)	1645 (85.5%)
Lobulillar	83 (7.8%)	127 (10.2%)	144 (9.7%)	160 (8.3%)
Mixed	12 (1.1%)	26 (2.1%)	25 (1.7%)	0 (0.0%)
Others	57 (5.4%)	32 (2.6%)	95 (6.4%)	120 (6.2%)
Unknown	0 (0.0%)	1 (0.1%)	0 (0.0%)	0 (0.0%)
Histological Grade				
1	72 (6.8%)	178 (14.3%)	125 (8.4%)	125 (6.5%)
2	446 (42.1%)	528 (42.4%)	568 (38.1%)	880 (45.7%)
3	490 (46.2%)	431 (34.6%)	638 (42.8%)	849 (44.1%)
Unknown	52 (4.9%)	109 (8.7%)	160 (10.7 %)	71 (3.7%)
Pathologic primary tumor size				
1	533 (50.3%)	532 (42.7%)	615 (41.2%)	1126 (58.5%)
2	498 (47.0%)	644 (51.7%)	776 (52.0%)	772 (40.1%)
3	29 (2.7%)	70 (5.6%)	100 (6.7%)	27 (1.4%)
Nodes Affected				
No	1058 (99.8%)	0 (0.0%)	0 (0.0%)	1923 (99.9%)
Yes	2 (0.2%)	1246 (100.0%)	1491 (100.0%)	2 (0.1%)
Unknown	0 (0.0%)	0 (0.0%)	0 (0.0%)	0 (0.0%)
ER				
Negative	418 (39.4%)	266 (21.3%)	553 (37.1%)	628 (32.6%)
Positive	641 (60.5%)	978 (78.5%)	931 (62.4%)	1293 (67.2%)
Unknown	1 (0.1%)	2 (0.2%)	7 (0.5%)	4 (0.2%)
PR				
Negative	466 (44.0%)	379 (30.4%)	581 (39.0%)	763 (39.6%)
Positive	590 (55.7%)	860 (69.0%)	825 (55.3%)	1160 (60.3%)
Unknown	4 (0.4%)	7 (0.6%)	85 (5.7%)	2 (0.1%)

Additional Table (cont): Characteristics of women enrolled in the four clinical trials

Variables		Study GEICAM/9805 N=1060	Study GEICAM/9906 N=1246	Study BCIRG001 N=1491	Study GEICAM/2003-02 N=1925
HER2					
	Negative	561 (52.9%)	849 (68.1%)	965 (64.7%)	1704 (88.5%)
	Positive	93 (8.8%)	276 (22.2%)	285 (19.1%)	181 (9.4%)
	Unknown	406 (38.3%)	121 (9.7%)	241 (16.2%)	40 (2.1%)
Type of Surgery					
	Mastectomy	480 (45.3%)	751 (60.3%)	883 (59.2%)	526 (27.3%)
	Conservative	580 (54.7%)	495 (39.7%)	608 (40.8%)	1399 (72.7%)
	Unknown	0 (0.0%)	0 (0.0%)	0 (0.0%)	0 (0.0%)
Chemotherapy					
	No*	9 (0.8%)	4 (0.3%)	11 (0.7%)	8 (0.4%)
	Yes	1051 (99.2%)	1242 (99.7%)	1480 (99.3%)	1917 (99.6%)
Hormonotherapy					
	None	299 (28.2%)	251 (20.1%)	393 (26.4%)	510 (26.5%)
	Yes	689 (65.0%)	995 (79.9%)	964 (64.7%)	1414 (73.5%)
	Unknown	72 (6.8%)	0 (0.0%)	134 (9.0%)	1 (0.1%)
Body Surface					
	1.16-1.59	234 (22.1%)	273 (21.9%)	262 (17.6%)	392 (20.4%)
	1.59-1.67	229 (21.6%)	293 (23.5%)	241 (16.2%)	378 (19.6%)
	1.67-1.76	213 (20.1%)	248 (19.9%)	292 (19.6%)	386 (20.1%)
	1.76-1.86	203 (19.2%)	228 (18.3%)	308 (20.7%)	385 (20.0%)
	1.86-2.92	178 (16.8%)	199 (16.0%)	388 (26.0%)	383 (19.9%)
	Unknown	3 (0.3%)	5 (0.4%)	0 (0.0%)	1 (0.1%)
	Mean (SD)	*1.72 (0.16)*	*1.71 (0.16)*	*1.76 (0.19)*	*1.71 (0.16)*
BMI					
	< 18.5	17 (1.6%)	3 (0.2%)	31 (2.1%)	16 (0.8%)
	18.5-24.9	419 (39.5%)	464 (37.2%)	601 (40.3%)	828 (43.0%)
	25.0-29.9	369 (34.8%)	452 (36.3%)	488 (32.7%)	643 (33.4%)
	30.0-34. 9	178 (16.8%)	223 (17.9%)	237 (15.9%)	313 (16.3%)
	≥ 35.0	74 (7.0%)	99 (7.9%)	134 (9.0%)	124 (6.4%)
	Unknown	3 (0.3%)	5 (0.4%)	0 (0.0%)	1 (0.1%)
	Mean (SD)	*26.9 (5.1)*	*27.2 (5.0)*	*26.9 (5.5)*	*26.6 (4.9)*
Adverse Events					
	Grade 3	215 (20.3%)	594 (47.7%)	440 (29.5%)	814 (42.3%)
	Grade 4	49 (4.6%)	128 (10.3%)	50 (3.4%)	151 (7.8%)
Undertreatment†					
	Epirubicine	-	82 (6.6%)	-	-
	Cyclophosphamide	44 (4.3%)	77 (6.2%)	50 (3.5%)	12 (0.6%)
	Fluorouracil	7 (1.4%)	77 (6.2%)	8 (1.1%)	11 (0.6%)
	Doxorubicin	24 (2.4%)	-	13 (0.9%)	13 (0.7%)
	Docetaxel	29 (5.8%)	-	51 (7.5%)	-
	Paclitaxel	-	89 (14.5%)	-	36 (4.4%)

* These women were randomized but did not received the corresponding treatment

† Defined as total dose received lower than 85% of the theoretical dose proposed in each of the trials and regimen arms
